# Nutraceuticals: Potential for Chondroprotection and Molecular Targeting of Osteoarthritis

**DOI:** 10.3390/ijms141123063

**Published:** 2013-11-21

**Authors:** Daniel J. Leong, Marwa Choudhury, David M. Hirsh, John A. Hardin, Neil J. Cobelli, Hui B. Sun

**Affiliations:** 1 Department of Orthopaedic Surgery, Albert Einstein College of Medicine and Montefiore Medical Center, Bronx, NY 10461, USA; E-Mails: daniel.leong@einstein.yu.edu (D.J.L.); marwa.choudhury@einstein.yu.edu (M.C.); dhirsh@montefiore.org (D.M.H.); johnhardin@arthritis.org (J.A.H.); ncobelli@montefiore.org (N.J.C.); 2 Department of Radiation Oncology, Albert Einstein College of Medicine and Montefiore Medical Center, 1300 Morris Park Avenue, Golding 101, Bronx, NY 10461, USA

**Keywords:** nutraceuticals, osteoarthritis, molecular targets

## Abstract

Osteoarthritis (OA) is a degenerative joint disease and a leading cause of adult disability. There is no cure for OA, and no effective treatments which arrest or slow its progression. Current pharmacologic treatments such as analgesics may improve pain relief but do not alter OA disease progression. Prolonged consumption of these drugs can result in severe adverse effects. Given the nature of OA, life-long treatment will likely be required to arrest or slow its progression. Consequently, there is an urgent need for OA disease-modifying therapies which also improve symptoms and are safe for clinical use over long periods of time. Nutraceuticals—food or food products that provide medical or health benefits, including the prevention and/or treatment of a disease—offer not only favorable safety profiles, but may exert disease- and symptom-modification effects in OA. Forty-seven percent of OA patients use alternative medications, including nutraceuticals. This review will overview the efficacy and mechanism of action of commonly used nutraceuticals, discuss recent experimental and clinical data on the effects of select nutraceuticals, such as phytoflavonoids, polyphenols, and bioflavonoids on OA, and highlight their known molecular actions and limitations of their current use. We will conclude with a proposed novel nutraceutical-based molecular targeting strategy for chondroprotection and OA treatment.

## 1. Introduction

Osteoarthritis (OA) affects over 27 million Americans, is a leading cause of pain and disability [1,2], and is a significant economic burden in the United States, with over $185.5 billion in annual medical care expenditure [3]. OA is a disease of the entire synovial joint, and affects the underlying bone, synovium, meniscus, ligaments/tendons, and articular cartilage [4,5]. Progressive degradation and eventual loss of articular cartilage is the pathological hallmark of osteoarthritis, and is a major target for exploring disease-modifying treatment [4,6–8]. Cartilage plays a major role in cushioning the ends of the bones, allowing for the articulation of opposing joint surfaces. Destruction of articular cartilage leads to bones rubbing against each other, causing stiffness, pain, and ultimately, loss of movement in the joints [9].

There is currently no cure for OA, and there are no therapies which slow or arrest OA progression [6,10]. So far, most treatments primarily focus on the secondary effects of the disease, such as relieving pain and improving joint function, but fail to address the evolving and complex nature of OA. For example, analgesics and nonsteroidal anti-inflammatory drugs (NSAIDs), which are commonly prescribed to OA patients, generally decrease pain and improve function, but have no demonstrated beneficial effect on chondroprotection or OA disease prevention and modification [11]. Furthermore, long-term use of available pharmacological agents to relieve OA symptoms is associated with substantial gastrointestinal, renal, and cardiovascular side effects [11,12]. Given the nature of OA, life-long treatment will likely be required to arrest or slow its progression. Consequently, there is an urgent need for OA disease-modifying therapies, which in the best case scenario also improve symptoms, and are safe for clinical use over long periods of time.

Nutraceuticals—food or food products that provide medical or health benefits, including the prevention and/or treatment of a disease—offer not only a safe alternative to current pharmacologic therapies, but may exert disease- and symptom-modification effects in OA [13]. Forty-seven percent of adults use non-prescribed alternative medications (including food supplements and nutraceuticals) for OA management [14]. Recent studies indicate phytoflavonoids, polyphenols, and bioflavonoids, which are natural compounds found in fruits, teas, spices, wine, and vegetables, have shown the most potential to modify OA disease and symptoms based on their anti-inflammatory and anti-catabolic actions, and protective effects against oxidative stress [15]. In this review, we will summarize the clinical effects and potential mechanisms of action of commonly used nutraceuticals for OA treatment. We will then focus on nutraceuticals such as phytoflavonoids, polyphenols, and bioflavonoids, which have strong *in vitro* and pre-clinical evidence for treating OA, but are not well studied in clinical trials. The review will conclude with a novel nutraceutical-based targeting approach which may be utilized to effectively prevent OA initiation or arrest or slow OA progression.

## 2. Efficacy and Mechanism of Action of Currently Used Nutraceuticals

Nutritional agents, which offer favorable safety profiles, have long-generated interest for their potential in disease modification. Dietary macronutrients, including proteins and amino acids, fatty acids (e.g., omega-3), vitamins, and certain minerals not only provide building blocks for biological processes, but have the potential to support and influence the structure and function of joints [16–18]. For example, increased consumption of Vitamin C, an antioxidant vitamin found in many fruits and vegetables, was associated with reduced risk of cartilage loss and OA progression for OA patients [19]. Conversely, not eating “healthy foods,” including those that are high in fat and sugar, may exacerbate the disease [18,20]. Collectively, ingredients in foods are essential for joint health and certain ingredients have a critical impact on altering OA initiation and progression. Nutraceuticals including herbal medicines such as *Boswellia serrata*, *Harpogophytum procumbens*, *Phytodolor*, Willow bark, and supplements such as Green-lipped mussel, glucosamines, chondroitin, collagen hydrolysate, lipids (avocado/soybean unsaponifiables), and essential fatty acids, are used for OA (Table 1). In particular, glucosamine and chondroitin sulphate are among the most common nutraceuticals used for the treatment of OA. Glucosamine, an aminosaccharide initially isolated from the chitin of shellfish, is an important component of glycosaminoglycan chains and the production of proteoglycans, a major cartilage extracellular matrix protein [21]. Chondroitin sulphate is a glycosaminoglycan used in the synthesis of proteoglycans [22]. Despite the large number of studies examining the efficacy of glucosamine, chondroitin sulphate, or the two in combination for the treatment of OA, studies tend to show that these drugs result in little improvement compared with placebo in both symptomatic and structural outcomes [23–25]. These clinical trial findings may be due to the complexity and challenge of OA treatment, in addition to the effectiveness of dose, route of administration, and quality of the various products. Furthermore, clearly understanding the mechanism of action of glucosamine and chondroitin sulfate may provide better guidance for clinical use.

## 3. Pre-Clinical and Clinical Effects of Phytoflavonoids, Polyphenols, and Bioflavonoids Nutraceuticals on OA

Recent studies suggest that nutraceutical compounds such as phytoflavonoids, polyphenols, and bioflavonoids, derived from green tea, pomegranate, ginger, turmeric and rose hips, have shown promising preliminary evidence for their chondroprotective effect in OA prevention and treatment (Table 2).

### 3.1. Green Tea

Green tea is one of the most commonly consumed beverages in the world and is a rich source of polyphenols including epigallocatechin 3-gallate (EGCG) [90]. EGCG has strong anti-oxidant activity, up to 25–100 times more potent than Vitamin C and E [91]. The efficacy of EGCG or green tea extracts in human arthritis has yet to be tested, but there is strong evidence in small animal studies for advancing green tea-based therapies toward clinical application.

EGCG administered to collagen-induced arthritis mice, an inflammatory model of arthritis, via drinking water, lowered arthritis incidence and slowed progression of disease [83]. This disease-modifying effect was associated with a decrease in inflammatory mediators TNF-α, COX-2, and lower levels of total immunoglobulins (IgG) and type II collagen-specific IgG levels, indicating a reduced inflammatory immune response [83]. Daily administration of green tea extracts in drinking water slowed progression of arthritis in rat adjuvant-induced arthritis, inhibited serum levels of IL-17, and increased serum levels of IL-10 [92]. As cartilage destruction is a hallmark of both OA and RA, and inflammation also plays a role in OA, albeit to a lesser extent than in RA, green tea extracts may exert a good potential for OA prevention and treatment.

### 3.2. Pomegranate

Pomegranate fruit is used in traditional medicines to treat inflammation and pain in diseases including arthritis [90]. Pomegranates are considered to have strong anti-oxidant properties due to their high content of soluble polyphenols hydrolyzable tannin and punicalagin [93]. Pomegranate is also rich in anthocyanins, a polyphenolic compound that exhibits anti-oxidant and anti-inflammatory capabilities [94]. In the mono-iodoacetate OA mouse model, pomegranate juice administered by oral gavage for two weeks significantly reduced cartilage damage and proteoglycan loss, especially in the groups receiving the higher doses [85]. This study provides some *in vivo* evidence that pomegranate juice may improve the joint pathology in OA.

### 3.3. Ginger

Ginger is a widely used condiment and has long been prescribed in China and India for conditions such as nausea, vomiting, headaches, and arthritis, due to its anti-inflammatory and circulatory stimulant effects [95,96]. Ginger is non-toxic and is generally recognized as safe by the United States Food and Drug Administration. As an alternative to NSAID therapy for arthritic conditions, ginger has shown moderately positive results [97]. A randomized, placebo-controlled, crossover study comparing ginger extracts and ibuprofen was performed and the study revealed significant improvement in symptoms for both groups before crossover. After the crossover, no difference was noted between the ginger- and placebo-treated groups [86]. A randomized, double-blind, placebo-controlled trial also studied the effects of ginger and galangal extracts, a spice that is closely related to ginger, in the treatment of knee OA. OA patients treated with ginger and galangal extracts showed greater improvement in pain compared to the placebo group [87].

### 3.4. Tumeric

Turmeric is a widely used spice and is generally regarded as safe [98]. The major component of turmeric is curcumin, which constitutes up to 90% of total curcuminoid content. Although curcumin has been demonstrated to exert potent anti-inflammatory effects *in vitro*, there is no clinical data available for the effect of curcumin in OA treatment [19]. However, OA patients treated with a formulation containing curcumin exhibited positive results in pain management and mobility compared to the placebo control [88].

### 3.5. Rosehip Powder

Rosehip powder is extracted from fruits of the rose plant, and has been used extensively in traditional medicine [99]. A meta-analysis of randomized controlled trials (RCTs) showed rosehip powder reduced pain and led to reduced use of analgesics in OA patients [89]. A longer-term clinical trial comparing different rosehip formulations in patients with knee OA is currently undergoing (Clinical trial NCT01430481).

## 4. Nutraceuticals for Molecular Targeting of OA

### 4.1. Molecules in Pathology of OA Initiation and Progression

Chondrocytes, the sole cell population within the articular cartilage, are primarily responsible for the maintenance of the extracellular matrix [100]. In healthy adult cartilage, chondrocytes are normally quiescent. However, in OA, chondrocytes undergo phenotypic alterations, which include abnormal proliferation, cell death, senescence, and significant changes in gene expression, such as increased expression of inflammatory cytokines, matrix proteins and proteolytic enzymes [4,101]. Together, these lead to a loss of homeostatic balance of the articular cartilage and osteoarthritis.

In the early stages of OA, many inflammatory mediators are expressed in the cartilage and synovial tissue, which contribute to the progression of the disease [102–104]. Increased inflammation is the consequence of many factors, including mechanical overloading, joint injury, adipose tissue, and cartilage matrix fragments [105,106]. Interleukin (IL)-1β and tumor necrosis factor (TNF)-α are considered the most prominent pro-inflammatory cytokines involved in OA [104]. Elevated levels of both IL-1β and TNF-α are found in OA joint tissues, including the articular cartilage, subchondral bone, synovial fluid and synovium [107]. IL-1β and TNF-α alter the homeostatic balance of chondrocytes by suppressing anabolic activity, stimulating catabolic breakdown of the articular cartilage, and increasing production of inflammatory mediators and reactive oxygen species (ROS). These effects of IL-1β and TNF-α are mediated, at least in part, by members of the mitogen-activated protein kinases (MAPK), nuclear factor-kappa (NF-κ) B transcription factors, and certain members of the Wnt-β-catenin signaling pathways [108–110]. IL-1β and TNF-α suppress expression of major structural components in the articular cartilage, including type II collagen and proteoglycans [111–114]. IL-1β and TNF-α also increase expression of proteolytic enzymes which directly cleave the cartilage matrix, including matrix metalloproteinases (MMPs)-1, −3, −13, and ADAMTS (a disintegrinlike and metalloproteinase with thrombospondin type 1 motifs) [115–117]. Furthermore, IL-1β and TNF-α stimulate production of inflammatory mediators prostaglandin E_2_ (PGE_2_) and cyclooxygenase 2 (COX-2), and ROS including nitric oxide (NO) and the superoxide anion [104,118].

Since the above-mentioned pro-inflammatory cytokines, inflammatory mediators, and proteolytic enzymes play critical roles in OA initiation and progression, these molecules have been targeted for OA treatment. Treatments against IL-1, such as Anakinra, a modified form of native IL-1Ra, have demonstrated chondroprotection in an animal model of OA [119], but its efficacy in human OA has not been clearly demonstrated [120]. Clinical trials of anti-TNF therapies are limited, and with mixed results [121–123]. Inhibiting the enzymes which directly cleave the cartilage matrix with MMP inhibitors have also been pursued as pharmacologic treatments. The use of MMP inhibitors in clinical trials, however, have resulted in severe musculoskeletal side effects including joint stiffness, inflammation, and pain, possibly due to their lack of specificity. MMPs are required for physiologic function in addition to the roles they play in OA [6,124]. Current efforts are now aimed at inhibition of specific MMPs, such as MMP-13 [125]. However, targeting only one molecule fails to address the broad and multimodal nature of OA, and may not effectively arrest or slow OA progression.

Recent studies suggest that ROS production induced by oxidative and other stresses may be a mediator of OA disease progression [126–128]. Elevated ROS production in conditions such as post-traumatic stress and aging may increase chondrocyte senescence and/or cell death [129,130]. Patients with knee OA exhibit higher levels of oxidative stress [131], and oxidative stress-induced damage [132]. Furthermore, levels of superoxide dismutase antioxidant enzymes are reduced in OA cartilage and joint fluid [133,134]. Boosting antioxidant defenses protects cartilage from traumatic impact-induced cartilage degradation and reduces OA severity in animal models of OA [135–137]. Upon further validation, these most recent progresses highly suggest that targeting altered response regulation against oxidative and other stresses should be a major criteria in developing effective nutraceutical-based products for OA prevention and treatment.

### 4.2. Molecular Targeting of OA by Nutraceuticals

The molecular targets of OA can be categorized as inflammatory, oxidative stress, or catabolic. These targets provide a significant rational foundation for pursuing nutraceuticals with anti-inflammatory, anti-catabolic activity, and anti-stress (*i.e.*, oxidative stress) properties for anti-OA nutraceutical drug selection and formulation. Nutraceuticals that have the potential to spontaneously target these aspects of OA may be the most druggable for molecular targeting of OA.

While OA is a complex disease with an unclear etiology and multiple risk factors, recent studies suggest the following are critical for OA initiation and disease progression: over activated catabolic activity mediated primarily by pro-inflammatory cytokines (*i.e.*, IL-1, TNF-α); deleterious stresses such as oxidative stress as well as the defense mechanisms against these stress factors (*i.e.*, oxidative stress); proteolytic enzymes which directly degrade the cartilage matrix such as matrix metalloproteinases, MMPs and aggrecanses, ADAMTS.

### 4.3. Anti-Inflammatory

Pomegranate extracts exert anti-inflammatory actions by inhibiting the activity of NF-κB, COX-2 and PGE_2_[94,138]. Prodelphinidin—a condensed polymeric tannin that can be found in pomegranate—inhibited PGE_2_ synthesis by down-regulating COX-2 in human chondrocytes [139]. Ginger extract has been demonstrated to decrease the IL-1β and LPS-induced production of NO and PGE_2_ in OA cartilage [140]. Furthermore, ginger extract was effective in inhibiting the production of TNF-α, PGE_2_, and COX-2 expression in human synoviocytes by regulating NF-κB activation and degradation of its inhibitor IkB-α [141]. It has also been reported to decrease the IL-1β-induced expression of TNF-α and TNF-α-induced production of COX-2 in synoviocytes [142]. Resveratrol is a polyphenolic phytoalexin present in grapes, berries, and peanuts. Resveratrol suppresses NF-κB-dependent pro-inflammatory products, including PGE_2_ and COX-2 [143,144]. Resveratrol has also been shown to inhibit IL-1β-induced apoptosis by inhibiting caspase-3 and downregulating the NF-κB pathway in chondrocytes [145]. Epigallocatechin 3-gallate (EGCG), a bioactive polyphenol found in green tea, inhibits the production of inflammatory mediators including PGE_2_, COX-2, and NF-κB [146]. By inhibiting the NF-κB pathway, EGCG suppressed IL6, IL-8, and TNF-α in IL-1β stimulated human OA chondrocytes [147]. Curcumin inhibited IL-1β-induced NF-κB activation and translocation, resulting in reduced expression of NF-κB downstream pro-inflammatory gene *COX-2* [90]. Curcumin also prevented production of NO, PGE_2_, IL-6, and IL-8 stimulated by IL-1β [96]. Rosehip preparations have anti-inflammatory properties, and have been shown to inhibit expression of iNOS and IL-1α, and IL-1β-induced IL-1α and IL-8 in chondrocytes. The combination of glucosamine and chondroitin sulfate suppressed gene expression of COX-2 and NF-κB induced by IL-1 in cartilage explants, leading to reduced production of NO and PGE_2_[148]. One of the mechanisms through which glucosamine or chondroitin sulfate exerts anti-inflammation is by inhibiting the IL-1β induced NF-κB pathway, resulting in a reduction in the COX-2 synthesis [149].

### 4.4. Anti-Oxidative Stress

Inflammatory cytokines (e.g., IL-1β and TNF-α) are known to stimulate chondrocytes and synoviocytes to produce high levels of oxygen free radicals [150]. Reactive oxygen species (ROS), which regulate many signaling pathways and pro-inflammatory cytokine gene activation, are important mediators in the pathogenesis of OA [151]. EGCG has been demonstrated to protect chondrocytes and other cell types from oxidative stress and ROS-mediated cytotoxicity [151–153]. EGCG pre-treatment of cells prevented H_2_O_2_-induced activation of MAPKs, suggesting EGCG has the potential to inhibit oxidative stress-mediated activation of inflammatory signaling pathways [154,155]. EGCG also increases innate antioxidant defenses, including expressions of catalase, superoxide dismutase, and glutathione peroxidase [155]. In addition, there is evidence that other nutraceuticals, such as ginger, may exert anti-oxidant effects [156]. The phenolic constituent of ginger, [6]-gingerol, inhibited LPS-induced iNOS expression and production of NO and other reactive nitrogen species in macrophages [157]. One of the components derived from pomegranate, anthocyanin, is a potent antioxidant, and has been reported to decrease lipid peroxidation and enhance activities of antioxidants catalase, superoxide dismutase, glutathione peroxidase and glutathione reductase in the liver [158,159].

### 4.5. Anti-Catabolic/Proteolytic Enzymes

Cartilage degradation, mainly caused by overactive catabolic activity primarily due to MMPs and ADAMTS, has been recognized as a major target for OA prevention and treatment. EGCG has been shown to significantly inhibit the expression and activities of proteolytic enzymes, including MMP-1 and MMP-13, and ADAMTS-1, −4, and −5 in chondrocytes [160,161]. Catabolic activity in other joint tissues, including synoviocytes and tendon, can also be suppressed by EGCG. EGCG suppressed TNF-α-induced production of MMP-1 and MMP-3 in RA synoviocytes and IL-1β-induced MMP-1, −3 and −13 expressions in human tendon fibroblasts [162]. Studies have also documented that EGCG increases anti-catabolic activity by inducing expression and activity of tissue inhibitors of MMPs (TIMP)-1 and −2 *in vitro* [163,164]. Curcumin exhibits an anti-catabolic effect by inhibiting MMP-3 and MMP-9 [96,165]. Curcumin also suppressed the release of proteoglycans in equine cartilage explants stimulated with IL-1β [166]. In other joint tissues, curcumin inhibited the IL-1β-induced production of MMP-1, MMP-9, and MMP-13 in tenocytes [167].

## 5. Conclusions

Nutraceuticals have been demonstrated to effectively suppress over activated inflammation and catabolic activity, and oxidative stress-induced deleterious responses. The suppression of inflammation and catabolic activity, in particular, are important properties of drugs targeting OA.

**Figure 1:**
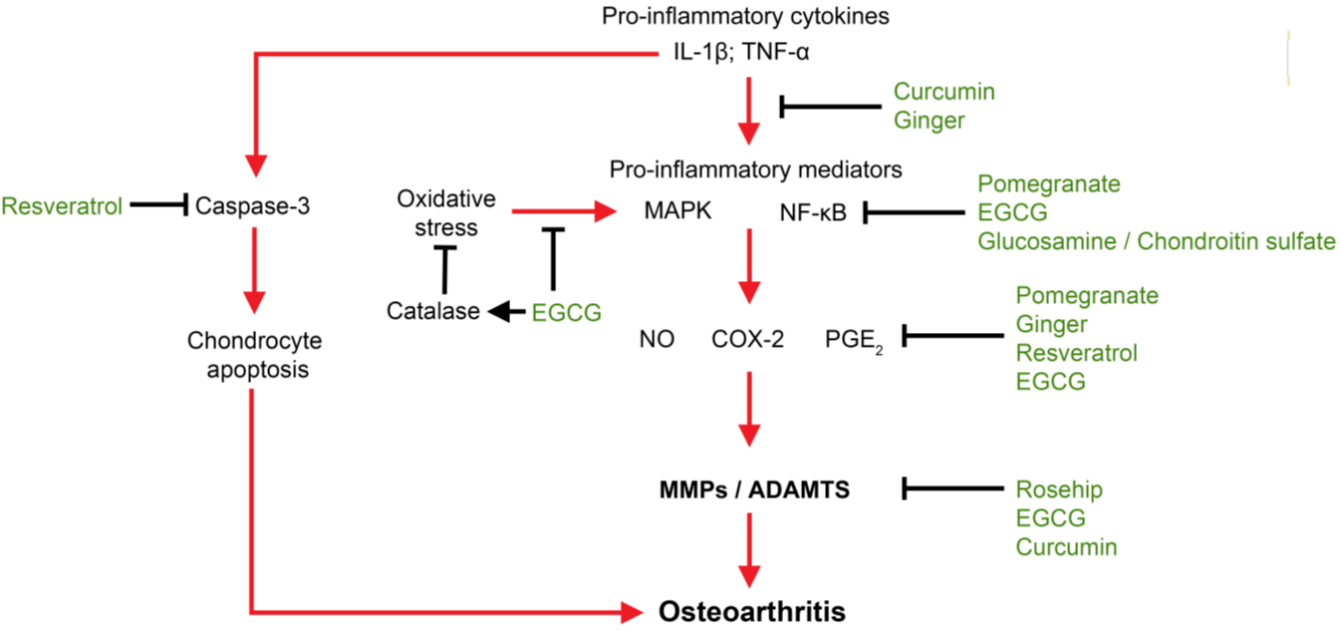
Molecular OA targeting of select nutraceuticals. Research findings support the concept that nutraceuticals can be used in a complementary manner to “shoot” multiple OA molecular targets.

Current pre-clinical and clinical trial data are promising, and show that individual nutraceutical compounds exert beneficial effects on OA, such as relieving pain and improving function. Their effects on disease modification have not yet been clearly demonstrated, or are still under investigation. Based on the effectiveness and actions of these nutraceutical compounds, efficacy of using an individual compound to treat a complex and chronic disease with multiple risk factors such as OA, may be limited. Future nutraceutical-based approaches may require a combination of compounds, and the selected compounds should: exert active effects on OA targets such as inflammation and catabolism, suppress oxidative stress and relieve chronic pain, as well as exerting complementary, additive, and/or synergistic anti-arthritic effects with other compounds within the formulation. These novel nutraceutical-based compound formulations which “shoot” many of the OA molecular targets (Figure 1) may serve as a therapeutic strategy for a new generation of nutraceuticals in OA prevention and treatment.

## Figures and Tables

**Table 1:** Clinical efficacy and mechanisms of action of commonly used nutraceuticals for osteoarthritis (OA).

Herbal/Plant-based extracts and medicines		
Nutraceuticals	Clinical efficacy	Mechanisms of action
*Boswellia serrata*	Relieved joint pain, reduced joint swelling and stiffness, increased joint flexion and walking distance [26–28]	Inhibited TNF-α-induced MMP-3 expression and protected against IL-1β-induced chondrocyte death [29]
Bromelain (pineapple extract)	Did not significantly relieve pain or quality-of-life symptoms [30]	Decreases PGE_2_ expression [31]
*Caesalpinia Sappan* extract (CSE)	Not reported	Inhibited inflammatory mediators IL-1β, iNOS, COX-2 and TNF-α expression in IL-1β stimulated primary human chondrocytes [32]. CSE also suppressed *MMP-1*, *MMP-3*, *MMP-7*, *MMP-9* and *MMP-13* gene expression [33]
Capsaicin	Reduced pain and stiffness and increased joint function [34–36]	Agonist for transient receptor potential vanilloid 1 (pain receptor); Prolonged exposure of capsaicin leads to desensitization of this pain pathway [37]
Cat’s claw	Reduced OA-associated pain [38,39]	Inhibit lipopolysaccharide (LPS)-induced PGE_2_ production and activation of TNF-α [38]
Chicory root	Improved pain and relieved joint stiffness [40]	Inhibits production of COX-2, iNOS, TNF-α, and NF-κB [41,42]
*Diallyl sulphide* (garlic extract)	Not reported	Inhibited IL-1β-induced expression of MMP-1, −3 and −13. Ameliorated OA in rabbit anterior cruciate ligament transaction mode and reduced MMP-1, −3, −13 [43]; Inhibited COX-2 expression induced by IL-1β [44]
Duhuo Jisheng Tang	Reduced pain and stiffness as well as improved physical function in OA patients [45]	Not reported
*Harpogophytum procumbens* (Devil’s claw)	Alleviates pain in OA patients [46–48]	Inhibited release of TNF-α, IL-1β, IL-6, and PGE2 [49]
*Phyllanthus emblica*	Not reported	Inhibited hyaluronidase and type II collagenase activities *in vitro* and reduced GAG release in cartilage explants from OA patients [50].
Willow bark	Reduced OA-related pain [51,52]	Not reported
**Supplements**		
Nutraceuticals	Clinical efficacy	Mechanisms of action
Aloe Vera	Protects against gastrointestinal effects of NSAIDs [53]	Not reported
Avocado/soybean unsaponifiables	Reduced pain in OA patients and reduced NSAID consumption [54,55]	Reduced levels of iNOS and MMP-13 [56]. Suppressed TNF-α, IL-1β, COX-2, and iNOS in LPS-activated chondrocytes [57]
Calcium Fructoborate	Not reported	Suppresses IL-1β, IL-6, iNOS *in vivo* [58]
Collagen hydrolysates	Alleviates OA-related pain [59,60]	Stimulate regeneration of type II collagen and increases biosynthesis of proteoglycans [59]
Edible Bird’s nest extract	Not reported	Reduced gene expression of MMP-1, MMP-3, IL-1, IL-6, IL-8, COX-2, PGE2, and iNOS and increased type II collagen, aggrecan and SOX-9 [61]
Genistein	Not reported	Reduces IL-1β and COX-2 protein synthesis in LPS-induced human chondrocytes [62].
Green-Lipped Mussel extract	Improved knee joint pain, stiffness and mobility [63]	Inhibits synthesis of pro-inflammatory molecule Leukotriene B4 and production of PGE2 [64]
Lactobacillus casei	Not reported	Decreased TNF-α, IL-6, NF-κB, COX-2, MMP-1, −3, −13 and increased IL-4 and IL-10 [65]
Methylsulfonylmethane (MSM)	Improved symptoms of pain and physical function [66]	Scavenge hydroxyl free radicals [67]; sulfur content rectifies dietary deficiencies of sulfur to improve cartilage formation [68]
Polyunsaturated fatty acids (PUFA)	High levels of N-3 PUFA associated with less cartilage loss [69]	N-3 PUFA abolished TNF-α, IL-1β, COX-2, MMP-3, −13, ADAMTS5 expression *in vitro* [70] and protected against cartilage degradation in OA prone animals [71]
*S*-adenosylmethionine	Reduced OA-related pain intensity from baseline [72–74]	Increases proteoglycan synthesis [75] and chondrocyte proliferation [76]
**Vitamins**		
Nutraceuticals	Clinical efficacy	Mechanisms of action
Niacinamide (B-complex vitamins)	Improved joint mobility [77]	Not reported
Vitamin C		Stimulates collagen and proteoglycan synthesis [78]
Vitamin D	No effect on pain severity or MRI-assessed quantitative cartilage loss [79]; Relieved OA-associated joint pain [80]	Not reported
Vitamin E	Relieved OA-related pain and improved physical function [81,82]	Not reported

Not reported: based on Pubmed search on 9/15/2013.

**Table 2:** The actions of select phytoflavonoids, polyphenols, and bioflavonoids nutraceuticals on arthritis.

Nutraceutical	Clinical effects	Preclinical effects
Green tea	Not reported	Lowered arthritis incidence and index score in collagen-induced arthritis [83] Decreased inflammatory mediators TNF-α, COX-2 [83] Reduced serum levels of IL-17, and increased serum levels of IL-10 [84]
Pomegranate	Not reported	Reduced cartilage damage and proteoglycan loss in OA mice [85]
Ginger	No difference between ginger-and placebo-treated groups in OA patients after 3 weeks [86] Improved pain in OA patients after 6 weeks [87]	Not reported
Tumeric	Improvement in pain and mobility [88]	Not reported
Rosehip powder	Reduced OA-associated pain [89]	Not reported
